# Early prediction of sepsis-induced coagulopathy in the ICU using interpretable machine learning: a multi-center retrospective cohort study

**DOI:** 10.3389/fmed.2025.1681621

**Published:** 2025-11-05

**Authors:** Tao Sha, Hao Jiang, Lei Feng

**Affiliations:** Department of Emergency, Huadong hospital, Fudan University, Shanghai, China

**Keywords:** sepsis-induced coagulopathy, machine learning, predictive models, early prediction, MIMIC-IV database

## Abstract

**Background:**

Sepsis-induced coagulopathy (*SIC*) is a fatal complication in ICU patients, yet early risk prediction remains challenging. This study aimed to develop an interpretable machine learning model for predicting *SIC* within seven days of ICU admission.

**Methods:**

Clinical data for model development were retrieved from the Medical Information Mart for Intensive Care-IV (MIMIC-IV) database. Feature selection was performed using three distinct algorithms: least absolute shrinkage and selection operator (LASSO) regression, random forest recursive feature elimination (RF-RFE), and the Boruta method. Ten machine learning models underwent training employing 5-fold cross-validation on the training subset, with subsequent evaluation on the validation subset encompassing discrimination, calibration, and clinical utility metrics. The optimal model underwent further interpretability analysis through SHapley Additive exPlanations (SHAP) to elucidate variable contributions and their directional effects. External validation was then conducted using the electronic Intensive Care Unit Collaborative Research Database (eICU-CRD). Finally, the best-performing model was implemented as a web-based Shiny application featuring an interactive interface.

**Results:**

Among 10,740 patients in MIMIC-IV, 2,232 (20.78%) developed *SIC* within 7 days post-ICU admission. A LightGBM model with thirteen variables demonstrated optimal performance, achieving an area under the receiver operating characteristic curve (AUROC) of 0.885 (95% confidence interval (CI): 0.874–0.897) in the internal validation set and 0.831 (95% CI: 0.819–0.843) in the external eICU-CRD cohort. Key predictive variables included Prothrombin Time-International Normalization Ratio (INR), platelet count, Sequential Organ Failure Assessment (SOFA), lactate, systolic blood pressure (SBP), red cell distribution width (RDW), bicarbonate, phosphate, hemoglobin, age, the presence of heart failure (HF), ischemic heart disease (IHD) and the use of continuous renal replacement therapy (CRRT). The model was deployed as a clinician-oriented web application providing an accessible interface (https://shatao.shinyapps.io/Sepsis_Induced_Coagulopathy/).

**Conclusion:**

This model demonstrated strong predictive ability and clinical interpretability, enabling early *SIC* identification and targeted intervention.

## 1 Introduction

Sepsis is a critical medical condition marked by systemic organ dysfunction due to an aberrant host response to infection, which involves immune dysregulation and subsequent multi-organ failure (1, 2). Heightened clinical awareness and early recognition are critical to enable timely administration of appropriate antibiotics and other urgent interventions, thereby improving patient outcomes (3, 4). A common complication of sepsis is *SIC*, a condition characterized by vascular endothelial damage and systemic coagulation abnormalities triggered by the septic process (5, 6). A secondary analysis of two European randomized controlled trials reported *SIC* prevalence rates of 22.1% (HYPRESS trial) and 24.2% (SISPCT trial) (7). Epidemiological studies indicate that coagulation disorders occur in approximately 50–70% of septic patients, with nearly 35% progressing to disseminated intravascular coagulation (DIC) (8, 9). The coagulation cascade becomes activated in sepsis primarily through tissue factor exposure on stimulated monocytes and vascular endothelial cells. This procoagulant response occurs because natural regulatory mechanisms, particularly tissue factor pathway inhibitor function, become inadequate during sepsis (8). Simultaneously, inflammatory mediators characteristic of sepsis suppress critical anticoagulant systems, most notably the protein C pathway. Concurrent overexpression of plasminogen activator inhibitor-1 (PAI-1) creates a dual defect - both promoting excessive fibrin deposition and inhibiting its normal clearance (8). These pathological alterations in hemostatic balance drive widespread microthrombosis, ultimately causing tissue hypoperfusion and contributing to sepsis-induced multiple organ failure. Emerging findings, including data from observational studies and large randomized controlled trials (RCTs), suggest anticoagulant therapy offers significant mortality reduction and clinical outcome improvement in septic patients with confirmed coagulopathy (10–12). However, in patients without coagulation abnormalities, the use of anticoagulants appears to confer no survival benefit while increasing the risk of bleeding complications, thus warranting cautious consideration in clinical practice (12, 13). Early identification of coagulopathy risk factors in septic patients enables timely diagnosis of *SIC*, while targeted therapeutic strategies addressing the underlying pathophysiology are essential for improving survival and clinical outcomes. Consequently, there is an urgent need for accurate, early prediction tools to identify septic patients at highest risk for developing *SIC*, enabling preemptive management.

The International Society of Thrombosis and Haemostasis (ISTH)’s Scientific and Standardization Committee (SSC) on Disseminated Intravascular Coagulation (DIC) in 2017 (5) established the *SIC* criteria, encompassing three key parameters: INR, platelet count, and composite SOFA score components. The scoring system was applied as follows: for INR values, scores of 0, 1, and 2 were assigned corresponding to ≤ 1.2, > 1.2, and > 1.4 respectively. Platelet counts ≥ 150 × 10^∧^9/L received 0 points, while counts < 150 × 10^∧^9/L and < 100 × 10^∧^9/L were assigned 1 and 2 points respectively. The composite SOFA score was derived from the sum of individual scores for respiratory, cardiovascular, hepatic, and renal systems, with each system component capped at a maximum of 2 points. A diagnosis of *SIC* required fulfillment of two conditions: first, the cumulative score from all three parameters (INR, platelet count, and composite SOFA) had to reach ≥ 4 points; Second, the combined score from just the coagulation parameters (INR and platelet count) needed to exceed 2 points.

Recent advances in machine learning have revolutionized predictive analytics in medicine by leveraging complex clinical datasets to forecast disease progression dynamically. Modern algorithms are particularly adept at capturing intricate, non-linear relationships between predictors and outcomes, making them well-suited for analyzing high-dimensional biomedical data (14, 15). This capability is crucial in the context of sepsis, where emerging evidence suggests that the associations between key physiological variables—such as serum osmolarity, bicarbonate levels, and others—and critical outcomes like mortality are often non-linear and cannot be fully characterized by traditional linear models (16, 17). This study aimed to develop and validate a machine learning-based framework for the early and dynamic prediction of *SIC*. Additionally, we sought to identify critical risk factors through interpretable modeling techniques to enhance clinical understanding of *SIC* pathogenesis.

## 2 Materials and methods

### 2.1 Data source

This study utilized data from two independent databases. Medical Information Mart for Intensive Care IV (MIMIC-IV, version 3.1) (18, 19) is a publicly available critical care database maintained by the Massachusetts Institute of Technology (MIT) that contains de-identified clinical data from Beth Israel Deaconess Medical Center (BIDMC), a tertiary academic hospital in Boston, United States. The database spans patient records from 2008 to 2022, encompassing 364,627 hospital admissions and 76,540 unique ICU stays across medical, surgical, cardiac, and neonatal intensive care units. The eICU-CRD is a multicenter repository containing de-identified clinical data from over 200,000 ICU admissions across the United States (2014–2015) (20). In this study, we utilized the MIMIC-IV cohort as a development dataset and the eICU-CRD cohort as an independent external validation dataset. Certification for the Collaborative Institutional Training Initiative (CITI) program was successfully completed by one author (Tao Sha), with issued credential ID 68314142, which is a prerequisite for accessing both the MIMIC-IV and eICU-CRD databases. The use of the MIMIC-IV database was approved by the Institutional Review Boards (IRB) of the Massachusetts Institute of Technology (MIT), and the requirement for informed consent was waived due to the de-identified nature of the data. Similarly, the creation of the eICU-CRD was approved by the IRB of MIT (Protocol No. 0403000206), and informed consent was waived for its original data collection. In accordance with institutional policies, we verbally notified the Ethics Committee of Huadong Hospital affiliated to Fudan University and received confirmation that formal ethics approval was not necessary. The study was reported according to the recommendations of the Transparent Reporting of a multivariable prediction model for Individual Prognosis Or Diagnosis (TRIPOD) statement (21).

### 2.2 Participants

Inclusion criteria: (1) Fulfilled Sepsis 3.0 diagnostic criteria (Society of Critical Care Medicine and European Society of Intensive Care Medicine in 2016); (2) Initial ICU admission.

**FIGURE 1:**
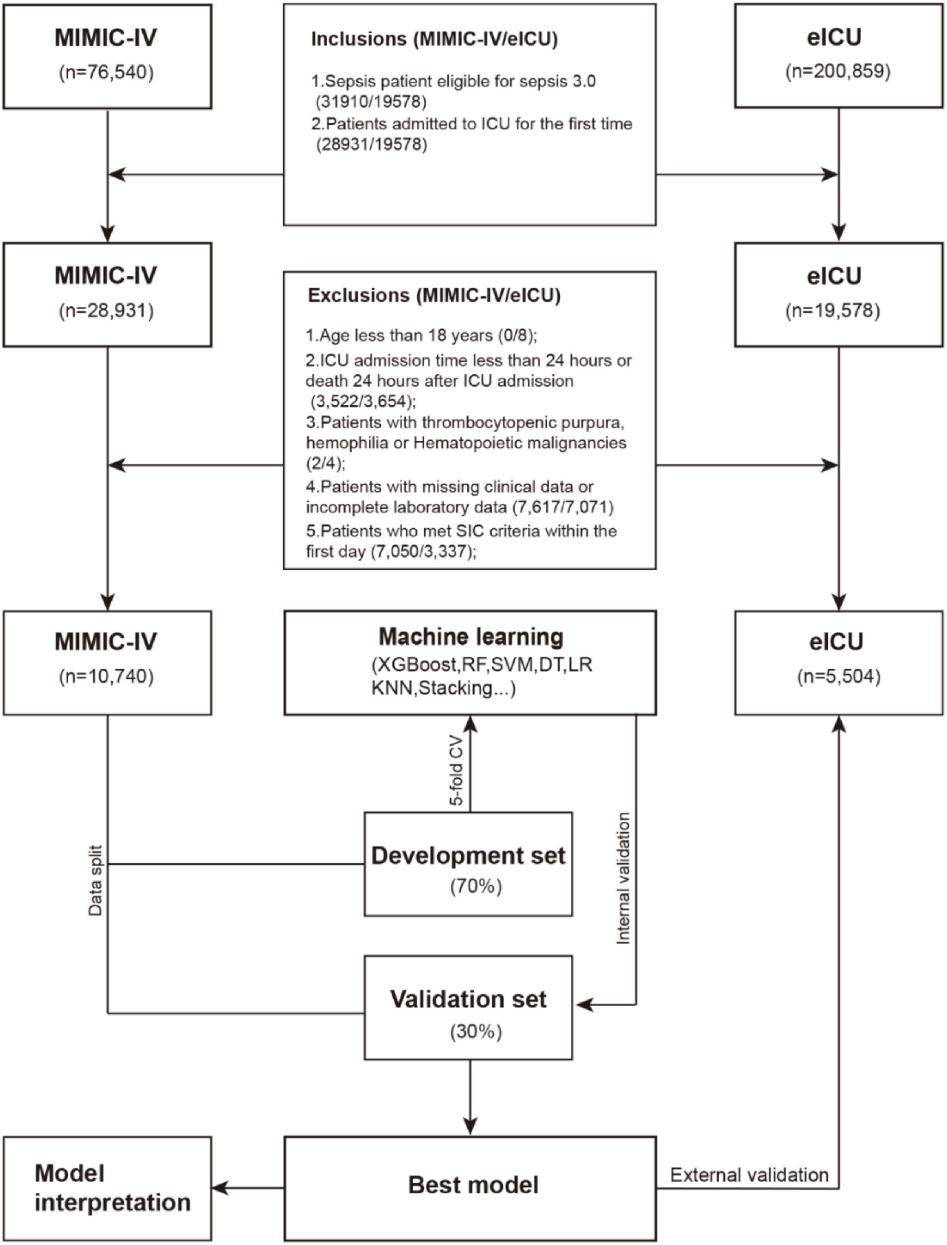
Workflow.

Exclusion criteria: (1) Under 18 years of age; (2) ICU stay duration < 24 h or mortality within 24 h post-admission; (3) Patients with thrombocytopenic purpura, hemophilia or hematopoietic malignancies; (4) Incomplete clinical or laboratory records; (5) Development of *SIC* within the first 24 h of ICU care. Figure 1 details the participant selection workflow.

### 2.3 Data extraction

Data extraction for ICU-admitted patients within the initial 24-h period was performed using PostgreSQL’s Structured Query Language (SQL) across both databases. Retrieved parameters included: (1) Demographic information: age, gender, and weight; (2) Underlying diseases: Hypertension (HTN), Cerebrovascular Accident (CVA), Chronic Kidney Disease (CKD), Cancer (CA), Diabetes Mellitus (DM), Hyperlipidemia (HLD), HF, IHD, Chronic Obstructive Pulmonary Disease (COPD). (3) Interventions: CRRT, ventilation. (4) Vital signs: heart rate (HR), respiratory rate (RR), SBP, diastolic blood pressure (DBP), temperature, percutaneous arterial oxygen saturation (SpO_2_); (5) Scores: SOFA, Simplified Acute Physiology Score II (SAPSII), Oxford Acute Severity of Illness Score (OASIS), Glasgow Coma Scale (GCS), Charlson Comorbidity Index (CCI). (6) Laboratory indicators: white blood cell (WBC), neutrophil, lymphocyte, hemoglobin, platelet count, RDW, chloride, potassium, sodium, magnesium, calcium, glucose, albumin, total cholesterol (TC), triglycerides (TG), lactate, partial pressure of carbon dioxide (pCO_2_), potential of hydrogen (pH), partial pressure of oxygen (pO_2_), d-dimer (DDI), fibrinogen, INR, prothrombin time (PT), alanine aminotransferase (ALT), aspartate aminotransferase (AST), direct bilirubin (DBil), total bilirubin (TBil), uric acid (UA), creatine kinase (CK), creatine kinase-MB (CKMB), n-terminal pro-brain natriuretic peptide (NTproBNP), troponin t (TNT), creatinine (Cr), blood urea nitrogen (BUN), bicarbonate, phosphate (PO4), central venous pressure (CVP).

Repeated measurements were aggregated as mean values over the initial 24-h ICU period. Prior to analysis, we implemented rigorous data quality control measures. Variables exhibiting > 20% missingness were excluded to ensure analytical robustness. For remaining variables with less than 20% missing data, we employed multiple imputation using a random forest approach (MissForest algorithm), which has demonstrated superior performance for clinical datasets compared to traditional imputation methods. Notably, the imputation was performed separately on training and validation sets to prevent leakage. This non-parametric method iteratively imputes missing values by modeling each variable as a function of other variables in the dataset, preserving complex relationships and interactions characteristic of critical care data. The imputation process was repeated for five iterations to ensure stability of estimates, with diagnostic checks confirming the preservation of original data distributions (Supplementary Figure 1). This study defined its primary outcome as new-onset *SIC* diagnosed during the first 7 ICU days using ISTH 2017 criteria.

### 2.4 Statistical analysis and model development

Statistical analyses were performed based on the characteristics of the data. For continuous variables, normality was assessed using the Shapiro–Wilk test. Data that followed a normal distribution were expressed as mean ± standard deviation (SD) and compared between groups using the *t*-test. Non-normally distributed continuous variables were summarized as median with interquartile ranges (IQR) and compared using the Mann–Whitney U test. Categorical variables were presented as counts (percentages, %) and compared between groups using the Chi-square test or Fisher’s exact test, as appropriate.

We performed a stratified partitioning of the MIMIC-IV dataset to ensure robust model evaluation. The complete cohort was randomly divided into a development set (70% of patients) for feature selection, model training, and hyperparameter optimization, and an internal validation set (30% of patients) reserved for interim performance assessment.

We employed a feature selection strategy to optimize predictive variables. First, LASSO regression was applied to identify parsimonious features through L1 regularization. Meanwhile, the Boruta algorithm (22), a random forest-based wrapper method, was implemented to detect all-relevant features by comparing original variables with permuted shadow features. Subsequently, variables were selected using the RF-RFE algorithm. The final feature subset was determined by taking the intersection of variables selected by all three methods, ensuring biological plausibility while maintaining statistical robustness.

Using this optimized feature set, we developed and compared 10 distinct machine learning models: logistic regression (LR), decision tree (DT), elastic net regression (Enet), light gradient boosting machine (LightGBM), K-nearest neighbors (KNN), random forest (RF), extreme gradient boosting (XGBoost), support vector machine (SVM), multilayer perceptron (MLP) and a stacked ensemble model (stacking). We employed Bayesian optimization with Gaussian processes for hyperparameter tuning, utilizing the expected improvement acquisition function over 100 iterations to identify parameter configurations maximizing the AUROC. Model performance was evaluated through stratified 5-fold cross-validation on the development set, with the optimal model for each algorithm selected based on peak AUROC performance. The optimal probability threshold for clinical deployment was determined using Youden’s index (J = sensitivity + specificity − 1) to balance classification metrics. All models underwent comprehensive evaluation on the internal validation set, assessing: (1) discrimination (AUROC); (2) classification metrics (F1-score, accuracy, recall/sensitivity, specificity); (3) calibration (Brier score, calibration curves); (4) clinical utility (decision curve analysis (DCA) across probability thresholds 0–100%). To evaluate the potential confounding effect of anticoagulant therapy, a sensitivity analysis was performed on the MIMIC-IV cohort. We excluded patients who received any anticoagulant medication (including Heparin Sodium, Enoxaparin Sodium, Dalteparin, Warfarin, Rivaroxaban, Apixaban, and Dabigatran Etexilate) during the first 24 h of ICU admission. The optimal model was then re-validated on this sub-cohort. Finally, the optimal model was subsequently validated on the external eICU-CRD cohort. Final model interpretability was achieved through SHAP. We generated a ranked feature importance plot based on mean absolute SHAP values and a swarm diagram to visualize the distribution of SHAP values across all samples, demonstrating both feature importance and effect directionality. We developed partial dependence plots (PDPs) for each selected feature to show marginal predictions while holding other variables constant. Case-specific SHAP value computations revealed feature importance variations across individual predictions, offering insights into the black-box nature of the model’s decision algorithm.

All statistical analyses were performed in R 4.4.3, and the tidymodels framework (version 1.3.0) was used for unified machine learning implementation. Two-sided *p*< 0.05 were considered statistically significant.

## 3 Results

### 3.1 Baseline characteristics

After applying inclusion/exclusion criteria, 10,740 patients from MIMIC-IV and 5,504 from eICU-CRD were analyzed. *SIC* developed in 2,232 (20.78%) and 1,175 (23.3%) cases, respectively, during the 7-day post-admission window.

Table 1 presents baseline characteristics of the MIMIC-IV study cohort. Compared with the non-*SIC* group, *SIC* patients exhibited significant demographic and clinical disparities. The *SIC* cohort was older (median age 71.0 vs. 67.0 years, *P* < 0.001), predominantly male (61.1% vs. 52.8%, *P* < 0.001), and had higher comorbidity burdens, including chronic kidney disease (25.8% vs. 15.6%, *P* < 0.001), heart failure (42.6% vs. 22.5%, *P* < 0.001), and ischemic heart disease (48.2% vs. 29.3%, *P* < 0.001). Clinically, *SIC* patients experienced worse outcomes: prolonged ICU stays (median 5.6 vs. 4.6 days, *P* < 0.001) and hospital stays (median 13.0 vs. 10.6 days, *P* < 0.001), higher 28-day in-hospital mortality (24.0% vs. 13.2%, *P* < 0.001) and 28-day ICU mortality (24.8% vs. 13.4%, *P* < 0.001). During initial ICU admission, *SIC* patients demonstrated significantly higher intervention requirements: mechanical ventilation (89.7% vs. 84.7%; *p* < 0.001) and continuous renal replacement therapy (14.4% vs. 3.3%; *p* < 0.001). Severity scores including SOFA (6.0 vs. 4.0), SAPSII (42.0 vs. 36.0), and CCI (6.0 vs. 5.0) were significantly elevated in *SIC* patients (all *P* < 0.001). Comparative analysis of laboratory profiles and vital signs demonstrated significantly depressed levels in *SIC* patients across multiple parameters: lymphocyte, hemoglobin, platelets count, calcium, albumin, total cholesterol, triglycerides, fibrinogen, bicarbonate, SBP and DBP. Conversely, they demonstrated elevated RDW, magnesium, glucose, lactate, DDI, PT, INR, ALT, AST, Dbil, Tbil, CK, NTproBNP, Cr, BUN, phosphate and CVP compared to patients without *SIC*.

**TABLE 1:** Patient baseline characteristics.

Variables	NON-*SIC* (*N* = 8,508)	*SIC* (*N* = 2,232)	*p*
**Demographic data**			
Height (cm)	168.7 ± 10.7	169.4 ± 10.5	0.011
Weight (kg)	79.0 (65.2, 95.0)	80.0 (67.5, 96.7)	0.002
Gender, n (%)			< 0.001
F	3,775 (47.2%)	843 (38.9%)	
M	4,225 (52.8%)	1,322 (61.1%)	
sAge (years)	65.5 ± 17.0	69.5 ± 14.5	< 0.001
**Underlying diseases**			
HTN			< 0.001
No	4,923 (57.9%)	1,438 (64.4%)	
Yes	3,585 (42.1%)	794 (35.6%)	
CVA			0.389
No	7,653 (90%)	2,022 (90.6%)	
Yes	855 (10%)	210 (9.4%)	
CKD			< 0.001
No	7,178 (84.4%)	1,656 (74.2%)	
Yes	1,330 (15.6%)	576 (25.8%)	
CA			< 0.001
No	7,367 (86.6%)	1,860 (83.3%)	
Yes	1,141 (13.4%)	372 (16.7%)	
DM			< 0.001
No	6,222 (73.1%)	1,488 (66.7%)	
Yes	2,286 (26.9%)	744 (33.3%)	
HLD			< 0.001
No	5,635 (66.2%)	1,302 (58.3%)	
Yes	2,873 (33.8%)	930 (41.7%)	
HF			< 0.001
No	6,594 (77.5%)	1,281 (57.4%)	
Yes	1,914 (22.5%)	951 (42.6%)	
IHD			< 0.001
No	6,011 (70.7%)	1,157 (51.8%)	
Yes	2,497 (29.3%)	1,075 (48.2%)	
COPD			< 0.001
No	7,146 (84%)	1,797 (80.5%)	
Yes	1,362 (16%)	435 (19.5%)	
**Clinical outcomes**			
ICU LOS (days)	4.6 (2.9, 8.9)	5.6 (3.3, 9.9)	< 0.001
Hospital day (days)	10.6 (6.5, 18.2)	13.0 (7.8, 21.2)	< 0.001
Death within hospital 28d			< 0.001
No	7,386 (86.8%)	1,697 (76%)	
Yes	1,122 (13.2%)	535 (24%)	
Death within ICU 28d			< 0.001
No	7,364 (86.6%)	1,679 (75.2%)	
Yes	1,144 (13.4%)	553 (24.8%)	
**Interventions**			
Ventilation			< 0.001
No	1,305 (15.3%)	231 (10.3%)	
Yes	7,203 (84.7%)	2,001 (89.7%)	
CRRT			< 0.001
No	8,227 (96.7%)	1,910 (85.6%)	
Yes	281 (3.3%)	322 (14.4%)	
**Scores**			
SOFA	4.8 ± 2.8	6.6 ± 3.3	< 0.001
SAPSII	37.5 ± 13.1	43.5 ± 13.6	< 0.001
OASIS	33.4 ± 8.1	35.3 ± 8.4	< 0.001
GCS	15.0 (13.0, 15.0)	15.0 (14.0, 15.0)	< 0.001
CCI	4.9 ± 3.0	6.1 ± 3.0	< 0.001
**Vital signs**			
HR (times/min)	87.0 (75.0, 101.0)	88.0 (75.0, 102.0)	0.384
SBP (mmHg)	124.9 ± 25.3	116.5 ± 25.0	< 0.001
DBP (mmHg)	68.0 (57.0, 81.0)	63.0 (54.0, 76.0)	< 0.001
SpO_2_ (%)	98.0 (95.0, 100.0)	98.0 (95.0, 100.0)	0.580
RR (times/min)	19.0 (16.0, 23.0)	19.0 (15.0, 23.0)	0.514
T (°F)	98.3 (97.7, 99.0)	98.2 (97.6, 98.8)	< 0.001
**Laboratory tests**			
Lym (10^9^/L)	1.1 (0.7, 1.7)	1.1 (0.6, 1.6)	< 0.001
WBC (10^9^/L)	12.0 (8.8, 16.0)	11.9 (8.2, 16.6)	0.287
Neu (10^9^/L)	9.6 (6.6, 13.8)	10.1 (6.5, 14.7)	0.144
Hb (g/L)	11.0 ± 2.2	10.7 ± 2.3	< 0.001
PLT (10^9^/L)	224.0 (179.0, 289.0)	170.0 (137.0, 228.0)	< 0.001
RDW (fL)	14.2 (13.3, 15.5)	14.6 (13.5, 16.1)	< 0.001
Cl (mmol/L)	104.0 (100.0, 108.0)	105.0 (100.0, 109.0)	0.134
K (mmol/L)	4.1 (3.7, 4.6)	4.2 (3.8, 4.7)	< 0.001
Na (mmol/L)	139.0 (136.0, 141.0)	139.0 (135.0, 141.0)	0.004
Mg (mmol/L)	1.9 (1.7, 2.2)	2.0 (1.8, 2.3)	< 0.001
Ca2 (mg/dL)	8.4 (7.9, 8.8)	8.2 (7.8, 8.7)	< 0.001
Glu (mmol/L)	132.0 (109.0, 170.0)	137.0 (112.0, 182.0)	< 0.001
Alb (g/dL)	3.1 ± 0.6	2.9 ± 0.6	< 0.001
TC (mg/dL)	152.0 (125.0, 185.0)	125.0 (103.0, 159.0)	< 0.001
TG (mg/dL)	147.0 (98.0, 230.0)	138.5 (89.0, 227.0)	0.055
Lac (mmol/L)	1.5 (1.1, 2.3)	1.9 (1.3, 2.9)	< 0.001
pCO_2_ (mmHg)	41.0 (36.0, 48.0)	42.0 (36.0, 48.0)	0.988
pH	7.4 (7.3, 7.4)	7.4 (7.3, 7.4)	< 0.001
pO_2_ (mmHg)	144.6 ± 110.5	150.4 ± 125.8	0.062
DDI (ng/mL)	1733.0 (900.0, 3888.5)	2312.0 (1203.0, 5982.0)	0.025
FIB (mg/dL)	367.0 (237.0, 584.0)	323.0 (214.0, 506.0)	< 0.001
INR	1.2 (1.1, 1.3)	1.3 (1.2, 1.4)	< 0.001
PT (s)	12.8 (11.9, 13.9)	14.0 (12.8, 15.4)	< 0.001
ALT (IU/L)	25.0 (15.0, 50.0)	28.0 (16.0, 58.0)	0.001
AST (IU/L)	34.0 (21.0, 62.0)	43.0 (25.0, 96.0)	< 0.001
DBil (mg/dL)	0.6 (0.2, 1.8)	1.2 (0.5, 2.6)	< 0.001
Tbil (mg/dL)	0.5 (0.3, 0.7)	0.6 (0.4, 1.0)	< 0.001
Cr (mg/dL)	0.9 (0.7, 1.4)	1.2 (0.8, 1.9)	< 0.001
BUN (mg/dL)	18.0 (13.0, 30.0)	24.0 (16.0, 41.0)	< 0.001
UA (mg/dL)	5.2 (2.9, 8.1)	6.0 (3.4, 9.1)	0.022
CK (IU/L)	170.0 (68.0, 521.0)	190.0 (68.0, 689.0)	0.087
CKMB (ng/mL)	5.0 (3.0, 10.0)	6.0 (3.0, 17.0)	< 0.001
NTproBNP (pg/mL)	2286.0 (717.5, 6717.0)	4262.0 (1307.0, 12171.0)	< 0.001
TNT (ng/mL)	0.1 (0.0, 0.3)	0.1 (0.0, 0.7)	< 0.001
HCO3 (mmol/L)	23.0 (20.0, 26.0)	22.0 (19.0, 24.0)	< 0.001
PO4 (mmol/L)	3.5 (2.8, 4.2)	3.7 (3.0, 4.7)	< 0.001
CVP (mmHg)	10.0 (7.0, 14.0)	11.0 (8.0, 15.0)	0.002

Statistical comparisons used *t*-tests (mean with standard deviation), Mann-Whitney tests (median with first and third quartiles), or chi-square/Fisher’s exact tests (number with percentage) based on variable distribution and type. HTN, Hypertension; CVA, Cerebrovascular Accident; CKD, Chronic Kidney Disease; CA, Cancer; T2DM, Type 2 Diabetes Mellitus; HLD, Hyperlipidemia; HF, Heart Failure; IHD, Ischemic Heart Disease; COPD, Chronic Obstructive Pulmonary Disease; LOS, Length of stay; CRRT, continuous renal replacement therapy; SOFA, Sequential Organ Failure Assessment; SAPSII, Simplified Acute Physiology Score II; OASIS, Oxford Acute Severity of Illness Score; GCS, Glasgow Coma Scale; CHARLSON, Charlson Comorbidity Index; HR, Heart Rate; SBP, Systolic Blood Pressure; DBP, Diastolic Blood Pressure; SpO_2_, Peripheral Oxygen Saturation; RR, Respiratory Rate; T, Temperature; WBC, White Blood Cell; Neu, Neutrophil; Lym, Lymphocyte; Hb, Hemoglobin; PLT, Platelet; RDW, Red Cell Distribution Width; Cl, Chloride; K, Potassium; Na, Sodium; Mg, Magnesium; Ca, Calcium; Glu, Glucose; Alb, Albumin; TC, Total Cholesterol; TG, Triglycerides; LAC, Lactate; PCO_2_, Partial Pressure of Carbon Dioxide; PH, Potential of Hydrogen; PO_2_, Partial Pressure of Oxygen; DDI, D-Dimer; FIB, Fibrinogen; INR, International Normalized Ratio; PT, Prothrombin Time; ALT, Alanine Aminotransferase; AST, Aspartate Aminotransferase; DBil, Direct Bilirubin; TBil, Total Bilirubin; UA, Uric Acid; CK, Creatine Kinase; CKMB, Creatine Kinase-MB; NT-proBNP, N-Terminal pro-Brain Natriuretic Peptide; TNT, Troponin T; Cr, Creatinine; BUN, Blood Urea Nitrogen; AB, Actual Bicarbonate; PO4, Phosphate; CVP, Central Venous Pressure.

This comprehensive analysis confirms that *SIC* patients represent a distinct high-risk subgroup with multisystem dysregulation, providing a foundation for subsequent predictive modeling.

### 3.2 Feature engineering

**FIGURE 2:**
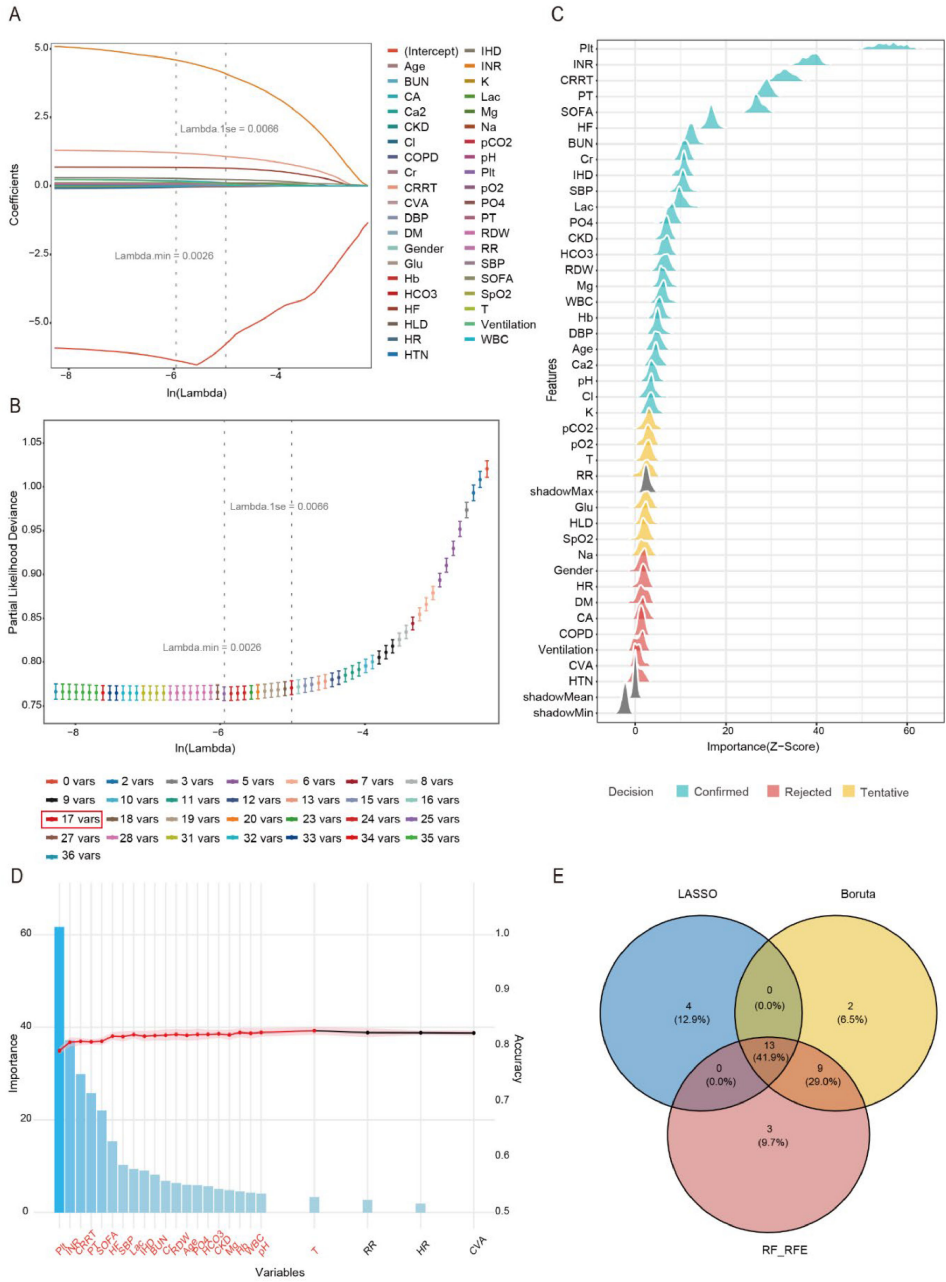
Feature engineering. **(A)** Variation characteristics of variable coefficients. **(B)** The process of selecting the optimal value of the parameter Lambda in the LASSO regression model is carried out by the cross-validation method. **(C)** Variable selection procedure using the Boruta algorithm. **(D)** Variable selection procedure using the RF-RFE. **(E)** Venn diagram illustrating the intersection of feature selection methods.

To identify the most predictive features for *SIC*, we employed a three-stage feature selection approach. We first applied LASSO regression with 10-fold cross-validation on the development set to penalize non-informative features. The optimal penalty parameter (the largest Lambda within 1 standard error of the minimum cross-validation error) was selected to balance model simplicity and generalizability (Figure 2A). LASSO retained 17 non-zero coefficient features (Figure 2B). We further applied the Boruta algorithm, a random forest-based wrapper method, to identify all potentially relevant features by comparing real features with permuted shadow features (Figure 2C), and Boruta selected 24 features with statistically significant importance (*p* < 0.01). Subsequently, we employed the RF-RFE with 5-fold cross-validation to identify the optimal 25-variable subset that maximized the AUROC, Figure 2D presents the feature selection results from RF-RFE, displaying a bar plot of variable importance scores for the final feature set (ranked by mean decrease in Gini index) and a line graph tracking AUC values across iterative feature subset sizes. Finally, the intersection of LASSO, Boruta and RFE yielded 13 clinically interpretable variables for model training: HF, IHD, CRRT, SOFA, PO4, Hb, Age, SBP, Plt, RDW, Lac, INR, HCO3. The intersection of selected features is visually presented in Figure 2E. This diagram shows the overlap in variables selected by the LASSO regression (17 variables), RF-RFE (25 variables), and the Boruta algorithm (24 variables). The numbers in each segment indicate the count of variables unique to each method and their overlaps. The final set of 13 predictive variables (in the central overlap) was derived from the intersection of all three methods.

### 3.3 Model performance comparisons

**FIGURE 3:**
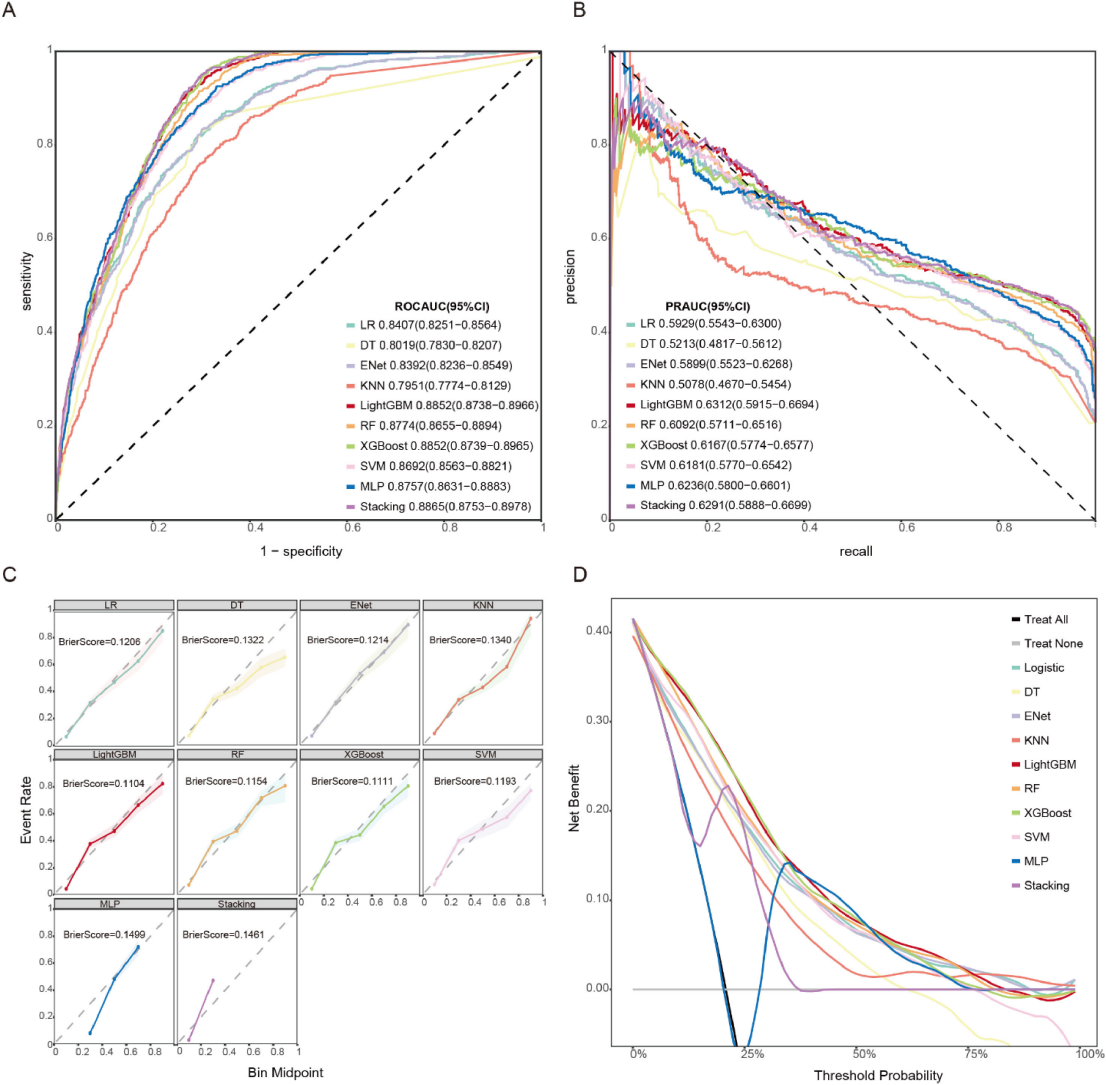
Model performances on the validation set. **(A)** ROC curve. **(B)** PR curve. **(C)** Calibration curve and Brier score. **(D)** DCA.

We systematically evaluated 10 machine learning algorithms across both training and validation cohorts, with comprehensive performance metrics for the validation set presented in Figure 3. The ROC and PR curves for all evaluated models on the training set are presented in Supplementary Figures 2, 3. LightGBM and stacking model exhibited the strongest overall performance, achieving the highest AUROC [0.885 (95% CI: 0.874–0.897) and 0.887 (95% CI: 0.875–0.898)] and area under the precision-recall curve (AUPRC) [0.631 (95% CI: 0.592–0.669) and 0.629 (95% CI: 0.589–0.670)] scores, along with exceptional NPV (0.962 and 0.969), while both demonstrated particularly high sensitivity (0.888 and 0.912) for identifying true positive cases. Tree-based ensemble methods, including XGBoost [AUROC: 0.885 (95% CI: 0.874–0.897)] and RF [AUROC: 0.877 (95% CI: 0.866–0.889)], consistently outperformed other approaches, showing 4.8–9.0% improvements in AUROC over traditional models like Logistic Regression (0.841) and ElasticNet (0.839). Although the MLP (AUROC: 0.876) and SVM (0.869) displayed intermediate performance, simpler models such as DT (0.802) and KNN (0.795) showed more limited discriminative ability, with KNN exhibiting notably lower sensitivity (0.672). The LightGBM model also demonstrated optimal calibration (Brier score 0.1104) and clinical utility, as evidenced by decision curve analysis (Figures 3C,D). While the stacking model showed comparable discrimination, its net benefit was marginally lower than LightGBM. KNN and DT exhibited poor clinical utility across threshold probabilities.

The comparative performance metrics of all 10 machine learning models are summarized in Table 2, which identified LightGBM, XGBoost, stacking, and RF as top performers based on AUROC (>0.88). LightGBM demonstrated superior discriminative ability (AUROC 0.885), the highest precision-recall performance (AUPRC 0.631), excellent calibration (Brier score 0.1104), and maximal clinical net benefit in decision curve analysis. Based on the model’s performance in terms of discrimination, calibration, and clinical applicability across both the training and validation datasets, we ultimately selected LightGBM as the optimal algorithm for subsequent external validation and model interpretation. The optimal probability threshold for clinical decision-making, determined by maximizing Youden’s index, was 0.232. The hyperparameter tuning process was conducted using Bayesian optimization with the complete optimization trajectory and final parameter configurations detailed in Supplementary Figure 4.

**TABLE 2:** Comparative evaluation metrics of predictive models in the validation set.

Models	AUROC	AUPRC	Accuracy	Sensitivity	Specificity	PPV	NPV
LightGBM	0.885	0.631	0.775	0.888	0.745	0.478	0.962
Stacking	0.887	0.629	0.770	0.912	0.733	0.473	0.969
XGBoost	0.885	0.617	0.768	0.890	0.736	0.469	0.962
RF	0.877	0.609	0.782	0.818	0.772	0.485	0.942
MLP	0.876	0.624	0.762	0.840	0.742	0.461	0.947
SVM	0.869	0.618	0.773	0.828	0.758	0.474	0.944
Logistic	0.841	0.593	0.729	0.816	0.706	0.421	0.936
ENet	0.839	0.590	0.730	0.815	0.708	0.423	0.936
DT	0.802	0.521	0.738	0.787	0.726	0.430	0.928
KNN	0.795	0.508	0.740	0.672	0.758	0.421	0.898

AUROC, area under the receiver operating characteristic curve; AUPRC, area under the precision-recall curve; PPV, positive predictive value; NPV, negative predictive value.

### 3.4 Sensitivity analysis

To assess the potential confounding effect of anticoagulant therapy on model performance, we conducted a sensitivity analysis on the MIMIC-IV cohort. We excluded patients who received any anticoagulant medication (including Heparin Sodium, Enoxaparin Sodium, Dalteparin, Warfarin, Rivaroxaban, Apixaban, and Dabigatran Etexilate) during the first 24 h of ICU admission. This process resulted in a sub-cohort of 8,335 patients. When our final LightGBM model was re-validated on this anticoagulant-free sub-cohort, it maintained strong predictive performance, achieving an AUROC of 0.857 (95% CI: 0.847–0.866). This result, which is highly comparable to the performance in the full cohort (AUROC: 0.885), indicates that the model’s predictive ability is robust and not substantially confounded by the early use of common therapeutic anticoagulants. The corresponding ROC curve and confusion matrix for this analysis are provided in Supplementary Figures 5, 6.

### 3.5 External validation

Our LightGBM model demonstrated excellent generalizability and robustness across different datasets. In the external validation using the eICU-CRD dataset, the model achieved an outstanding AUROC of 0.831 (95% CI: 0.819–0.843), confirming its strong predictive performance. The detailed ROC curve is presented in Supplementary Figure 7, while Supplementary Figure 8 shows the corresponding confusion matrix, further validating the model’s clinical applicability. These results highlight the model’s reliability for potential clinical implementation despite variations in data sources.

### 3.6 Interpretability analysis

**FIGURE 4:**
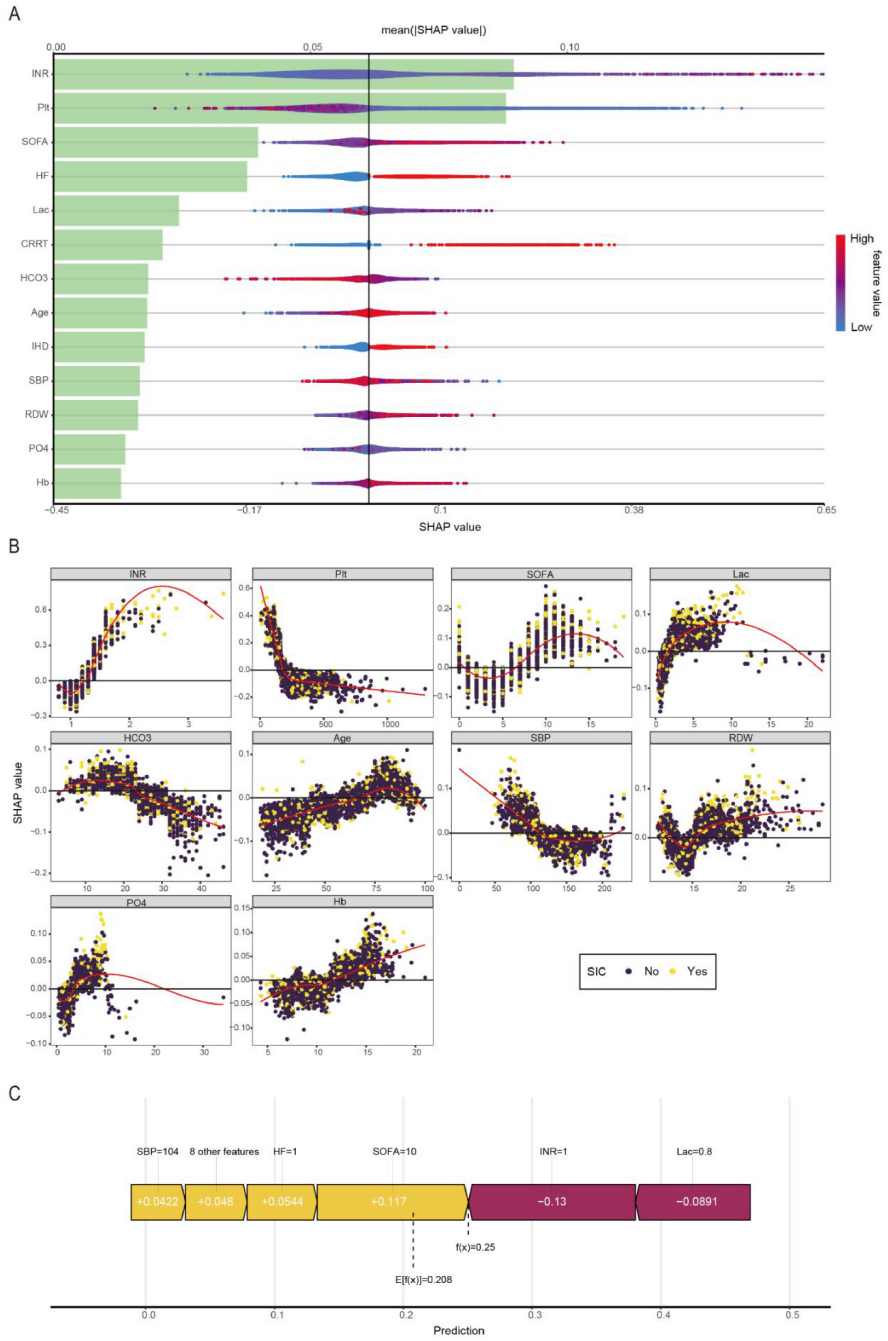
Visually interpret machine learning models using SHAP. **(A)** SHAP summary plot and feature importance plot. **(B)** SHAP dependency plots. **(C)** SHAP force plot.

Figure 4A presents an integrated visualization combining a SHAP beeswarm plot and feature importance ranking for the LightGBM model, where the lower x-axis represents raw SHAP values and the upper x-axis shows mean absolute SHAP values, with variables vertically ordered by descending importance. Figure 4B presents a partial dependence plot analysis of 10 continuous variables associated with the outcome. The analysis revealed significant clinical correlations with *SIC* development, demonstrating strong associations with elevated INR, thrombocytopenia, higher SOFA scores, increased lactate levels, pre-existing HF and IHD, requirement for CRRT, decreased bicarbonate levels, advanced age, hypotension, elevated RDW, hyperphosphatemia, and abnormal hemoglobin levels. Figure 4C presents an exemplary case analysis demonstrating the model’s risk prediction mechanism for an individual patient. The visualization employs a dual-color coding system: yellow represents risk-enhancing factors (positive SHAP values), while purple indicates protective factors (negative SHAP values). The magnitude of each feature’s contribution is quantified by its corresponding SHAP value [f(x)]. Notably, this case demonstrates that our LightGBM model predicted an elevated risk of *SIC* compared to the baseline population risk. The visualization provides clinicians with interpretable feature contributions for *SIC* risk stratification in septic patients, facilitating early prediction of clinical deterioration and data-driven therapeutic decision-making.

### 3.7 Model deployment

For practical clinical use and to expedite bedside decisions, we operationalized our optimized model via an interactive Shiny app deployed online.^1^ This accessible platform empowers clinicians to enter relevant patient data and instantly assess *SIC* risk. Crucially, the tool also deconstructs the prediction, showing the individual impact of each clinical characteristic on the risk score, thereby furnishing essential understanding of the underlying predictive factors.

## 4 Discussion

To our knowledge, this is the first interpretable machine learning model constructed based on the MIMIC-IV and eICU-CRD databases for the early prediction of *SIC* within 7 days of ICU admission. Our final LightGBM model, incorporating only 13 clinically relevant variables, provides an accurate, robust, and user-friendly tool for early *SIC* risk assessment.

Artificial intelligence has progressed rapidly, compared to conventional statistical approaches, ML techniques are capable of processing complicated, high-dimensional data with superior predictive accuracy. However, many studies either employ only a single algorithm for modeling, rely solely on the AUROC for performance evaluation, or utilize an excessive number of features at the expense of clinical practicality (23–26). Additionally, some are limited by their single-center design, which may compromise model generalizability. In contrast, our study adopted the Boruta, RF-RFE and LASSO algorithms for feature selection, constructed models using 10 distinct machine learning algorithms, and evaluated performance through multiple metrics, including ROC curves, calibration curves, Brier scores, and DCA. Ultimately, we developed a clinically practical LightGBM model incorporating only 13 variables.

LightGBM, an advanced gradient boosting decision tree (GBDT) framework developed by Microsoft Research, has gained prominence in machine learning due to its computational efficiency, optimized memory usage, and parallel processing capabilities (27). In medical classification tasks, its distinctive histogram-based algorithm, combined with Gradient-based One-Side Sampling (GOSS) and Exclusive Feature Bundling (EFB) techniques, enables rapid processing of high-dimensional biomedical data (e.g., genomic profiles, medical images, or EHRs) while maintaining superior classification accuracy. Three key advantages make it particularly valuable for clinical applications: (1) native handling of missing values and class imbalance—common challenges in real-world medical datasets; (2) scalable feature selection for identifying critical biomarkers from high-throughput data; and (3) significantly faster training speeds compared to conventional algorithms like XGBoost (28), which is crucial for time-sensitive healthcare decisions (e.g., early disease risk stratification).

SHAP, as an interpretable machine learning approach, provides insights into model predictions by quantifying feature contributions (29). In our study, the INR emerged as the most critical predictor for *SIC* development. Both platelet count and SOFA score also demonstrated significant predictive value. Previous studies have established INR as a moderately effective diagnostic tool for septic shock and sepsis, as well as a reliable prognostic marker for 30-day all-cause mortality. Specifically, an INR > 1.5 has been associated with increased mortality risk in septic patients (30). Sepsis-associated coagulopathy may progress from early-stage *SIC* to late-stage disseminated intravascular coagulation (DIC), with platelet counts below 50 × 10^9^/L often indicating DIC and poor prognosis. During sepsis, hemostatic balance is profoundly disrupted, characterized by concurrent coagulation activation and anticoagulation suppression. However, conventional laboratory markers (e.g., thrombocytopenia, prolonged PT, elevated fibrin degradation products, and hypofibrinogenemia) typically manifest only in advanced stages (31). In contrast, our LightGBM model demonstrates that platelet count and INR can predict *SIC* during the early, reversible phase of coagulopathy. There are some reasons that may explain INR’s superior predictive value in our model: First, INR provides a comprehensive assessment of coagulation status by integrating not only platelet quantity but also the synthesis and functionality of multiple coagulation factors, offering a more holistic evaluation than isolated parameters. Second, as a standardized derivative of PT, INR demonstrates enhanced sensitivity for detecting early coagulopathy compared to platelet counts alone, enabling earlier identification of coagulation abnormalities. Third, INR measurements exhibit greater reliability as they are less susceptible to common clinical confounders such as medication interference or transfusion effects that frequently impact platelet count accuracy. This combination of comprehensive coagulation profiling, early detection capability, and reduced vulnerability to confounding variables establishes INR as an optimal predictor in our *SIC* risk assessment model. These findings collectively position INR as an optimal early warning biomarker for *SIC* in septic patients.

Elevated lactate levels demonstrate a significant pathophysiological connection with *SIC* through two distinct mechanistic pathways. The primary mechanism involves lactate-induced endothelial injury, where increased circulating lactate concentrations directly compromise endothelial integrity and enhance vascular permeability (32, 33). This endothelial dysfunction subsequently triggers activation of the extrinsic coagulation cascade, ultimately contributing to systemic coagulopathy. The secondary mechanism relates to lactate-associated metabolic acidosis, which has been shown to impair thrombin production. These combined coagulation abnormalities may promote microvascular thrombosis, exacerbating tissue hypoperfusion and potentially creating a vicious cycle of worsening circulatory compromise.

RDW is an indicator reflecting the variability in RBC volume. Existing studies have confirmed its association with clinical outcomes in critically ill patients (34, 35). Similar findings have been observed in septic patients. For instance, a meta-analysis encompassing 11 studies demonstrated that elevated RDW was positively associated with mortality in sepsis patients (HR 1.14, 95% CI 1.09–1.20, *p* < 0.001), with findings robust across subgroups and sensitivity analyses (36). The potential mechanisms linking RDW to *SIC* may involve the following pathways: First, under conditions of inflammation and oxidative stress, accelerated erythrocyte destruction leads to excessive adenosine diphosphate (ADP) release into circulation, promoting platelet activation, adhesion, and aggregation. Second, sepsis disrupts iron metabolism and suppresses bone marrow hematopoiesis and megakaryocyte function, resulting in thrombocytopenia, elevated RDW, and subsequent coagulopathy (37, 38). RDW variability may thus reflect the degree of bone marrow suppression and indirectly indicate *SIC* risk. Third, inflammatory cytokines compromise vascular endothelial integrity, while sustained inflammation alters erythrocyte membrane glycoproteins and ion channel structures (39, 40), impairing erythrocyte deformability. These pathological changes exacerbate endothelial injury, trigger tissue factor release, and ultimately activate coagulation cascades, culminating in *SIC*. Therefore, within our model, RDW serves as a powerful proxy for the overall severity of the septic insult, which inherently encompasses the risk of developing *SIC*.

Elevated serum phosphate levels consistently correlate with increased mortality in sepsis, potentially attributable to phosphate-mediated cytotoxicity, diminished muscular function, vascular calcification, and cardiovascular pathology (41–44). Elevated serum phosphate levels may exacerbate microvascular dysfunction through multiple mechanisms, including inflammation, oxidative stress, and vascular calcification. The pivotal role of cardiovascular impairment in sepsis pathogenesis suggests septic patients possess unique biological vulnerability to hyperphosphatemia’s adverse consequences.

The present study offers several distinct strengths and clinical advantages over existing *SIC* prediction models. While a recent multi-center study by Tan et al. also developed an interpretable machine learning model for *SIC* prediction, their cohort was comparatively limited (*n* = 847) and their optimal model achieved an AUROC of 0.784 (95% CI: 0.711, 0.857) (45). In contrast, to our knowledge, our work presents the first interpretable machine learning model developed and externally validated on large-scale multicenter databases (MIMIC-IV, *n* = 10,740 and eICU-CRD) for the early prediction of *SIC* within 7 days of ICU admission, demonstrating superior discriminative performance (AUROC: 0.885 internally and 0.831 externally). The model’s clinical value is threefold. First, it enables the identification of high-risk patients days before the onset of *SIC*, providing a critical window for targeted interventions, such as intensified monitoring or careful consideration of anticoagulant therapy. Second, the model’s high performance (AUROC: 0.885 internally and 0.831 externally) is achieved using only 13 routinely available clinical variables, ensuring immediate practicality at the bedside without the need for specialized testing. Finally, and crucially, the integration of SHAP analysis transforms the model from a black box into a clinically interpretable tool. It not only provides a risk score but also elucidates the individualized contribution of key drivers (e.g., elevated INR, low platelet count) for each prediction, empowering clinicians with actionable insights for data-driven decision making. The robustness of our model is further affirmed by its stable performance in sensitivity analysis after excluding patients on anticoagulants. We have operationalized these advantages through an openly accessible web application, bridging the gap between advanced predictive analytics and frontline clinical practice.

This study also has several limitations. First, the retrospective nature of this investigation introduces potential selection and information biases; large-scale prospective validation studies remain essential to confirm predictor robustness across diverse populations. Second, our study did not adjust for medications that influence coagulation (e.g., aspirin, heparin) or evaluate their potential effects on *SIC* progression. Additionally, several advanced coagulation biomarkers, including tissue plasminogen activator-inhibitor complex (tPAI-C), thrombin-antithrombin complex (TAT), thromboelastography (TEG) parameters and antithrombin III (AT III) could not be incorporated into the model due to data unavailability in the database. Third, our predictive model was based only on static baseline measurements and did not capture dynamic changes in coagulation parameters over time. Future studies should develop time-series models incorporating serial measurements of PLT, INR, and other relevant biomarkers to improve the temporal prediction of *SIC* progression. Despite these limitations, our LightGBM model demonstrates potential as a clinically deployable tool for timely *SIC* identification.

## 5 Conclusion

We developed and validated a LightGBM model for early prediction of sepsis-induced coagulopathy (*SIC*) within 7 days of ICU admission. By incorporating SHAP analysis, our approach achieves both high predictive accuracy and transparent interpretation of key clinical features driving *SIC* pathogenesis. The model’s deployment as an interactive Shiny application (see text footnote 1) bridges advanced artificial intelligence with clinical decision-making at the bedside.

## Data Availability

The original contributions presented in the study are included in the article/Supplementary material, further inquiries can be directed to the corresponding author.

## Footnotes

1
https://shatao.shinyapps.io/Sepsis_Induced_Coagulopathy/
