# Supplementary material for: Early prediction of sepsis-induced coagulopathy in the ICU using interpretable machine learning: a multi-center retrospective cohort study
Source: Front Med (Lausanne). 2025 Nov 5;12:1681621. doi: 10.3389/fmed.2025.1681621 (PMC12626953; doi:10.3389/fmed.2025.1681621)
Supplement: Supplementary file 1 [file Data_Sheet_1.docx]

**Supplementary**

**Fig.S1 Missing data** HTN: Hypertension; CVA: Cerebrovascular Accident; CKD: Chronic Kidney Disease; CA: Cancer; T2DM: Type 2 Diabetes Mellitus; HLD: Hyperlipidemia; HF: Heart Failure; IHD: Ischemic Heart Disease; COPD: Chronic Obstructive Pulmonary Disease; LOS: Length of stay; CRRT: continuous renal replacement therapy; SOFA: Sequential Organ Failure Assessment; SAPSII: Simplified Acute Physiology Score II; OASIS: Oxford Acute Severity of Illness Score; GCS: Glasgow Coma Scale; CHARLSON: Charlson Comorbidity Index; HR: Heart Rate; SBP: Systolic Blood Pressure; DBP: Diastolic Blood Pressure; SpO2: Peripheral Oxygen Saturation; RR: Respiratory Rate; T:Temperature; WBC: White Blood Cell; Neu: Neutrophil; Lym: Lymphocyte; Hb: Hemoglobin; PLT: Platelet; RDW: Red Cell Distribution Width; Cl: Chloride; K: Potassium; Na: Sodium; Mg: Magnesium; Ca: Calcium; Glu: Glucose; Alb: Albumin; TC: Total Cholesterol; TG: Triglycerides; LAC: Lactate; PCO2: Partial Pressure of Carbon Dioxide; PH: Potential of Hydrogen; PO2: Partial Pressure of Oxygen; DDI: D-Dimer; FIB: Fibrinogen; INR: International Normalized Ratio; PT: Prothrombin Time; ALT: Alanine Aminotransferase; AST: Aspartate Aminotransferase; DBil: Direct Bilirubin; TBil: Total Bilirubin; UA: Uric Acid; CK: Creatine Kinase; CKMB: Creatine Kinase-MB; NT-proBNP: N-Terminal pro-Brain Natriuretic Peptide; TNT: Troponin T; Cr: Creatinine; BUN: Blood Urea Nitrogen; AB: Actual Bicarbonate; PO4: Phosphate; CVP: Central Venous Pressure.

**
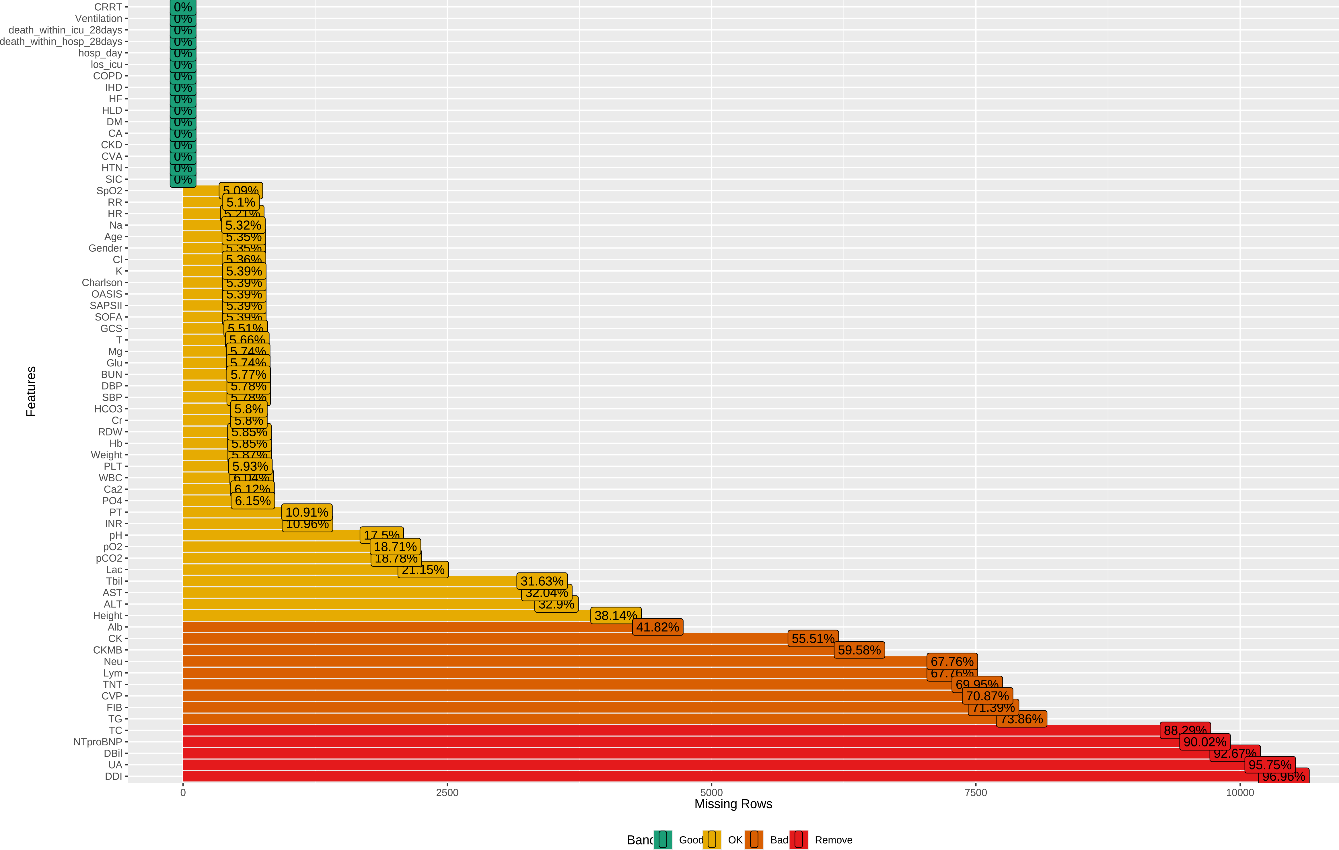
**

**Fig.S2 Receiver Operating Characteristic (ROC) Curve on training set**

**
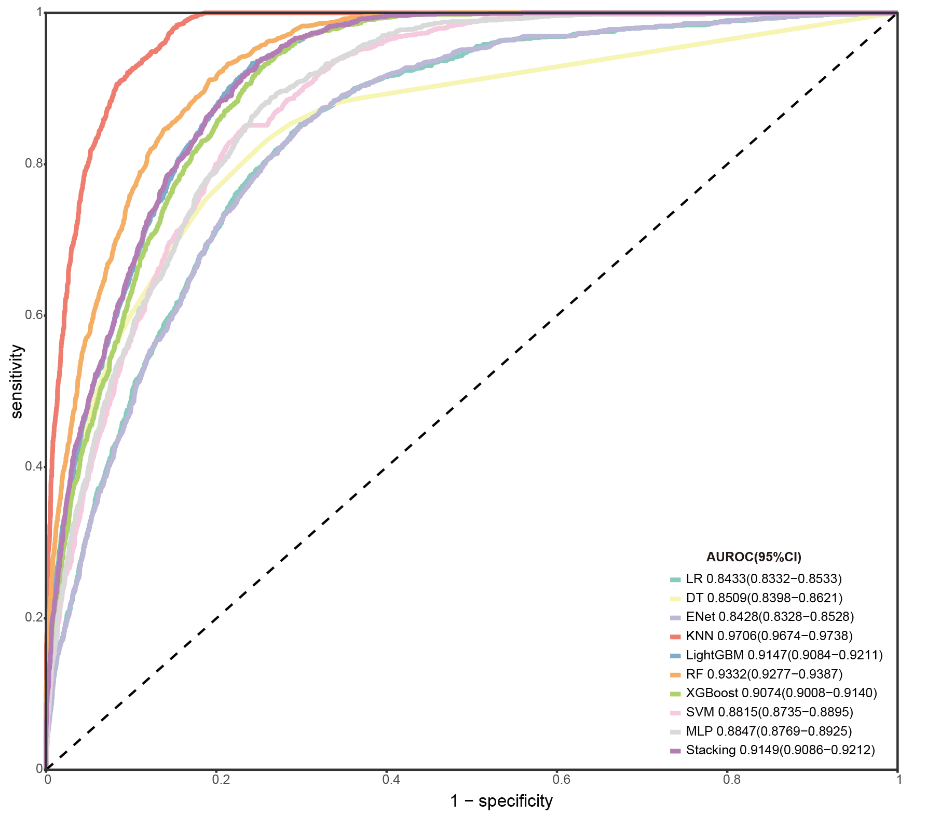
**

**Fig.S3 Precision-Recall (PR) curve**

**
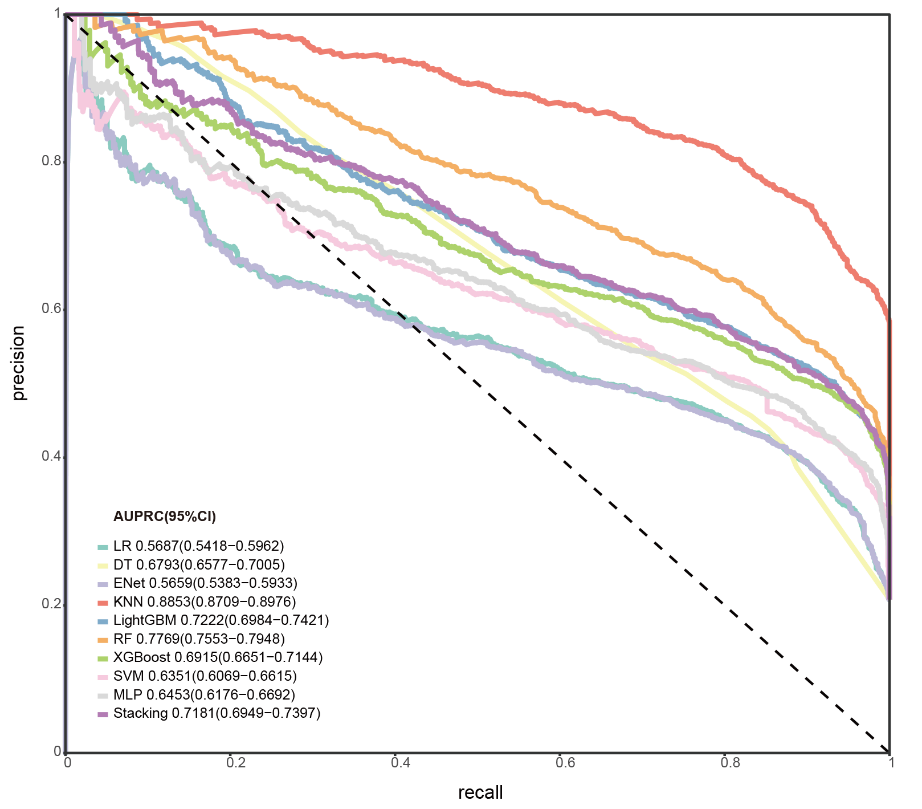
**

**Fig.S4 Hyperparameter Optimization for LightGBM Model**

mtry=3, trees=1777, min_n=23, tree_depth=11, learn_rate=0.001

**
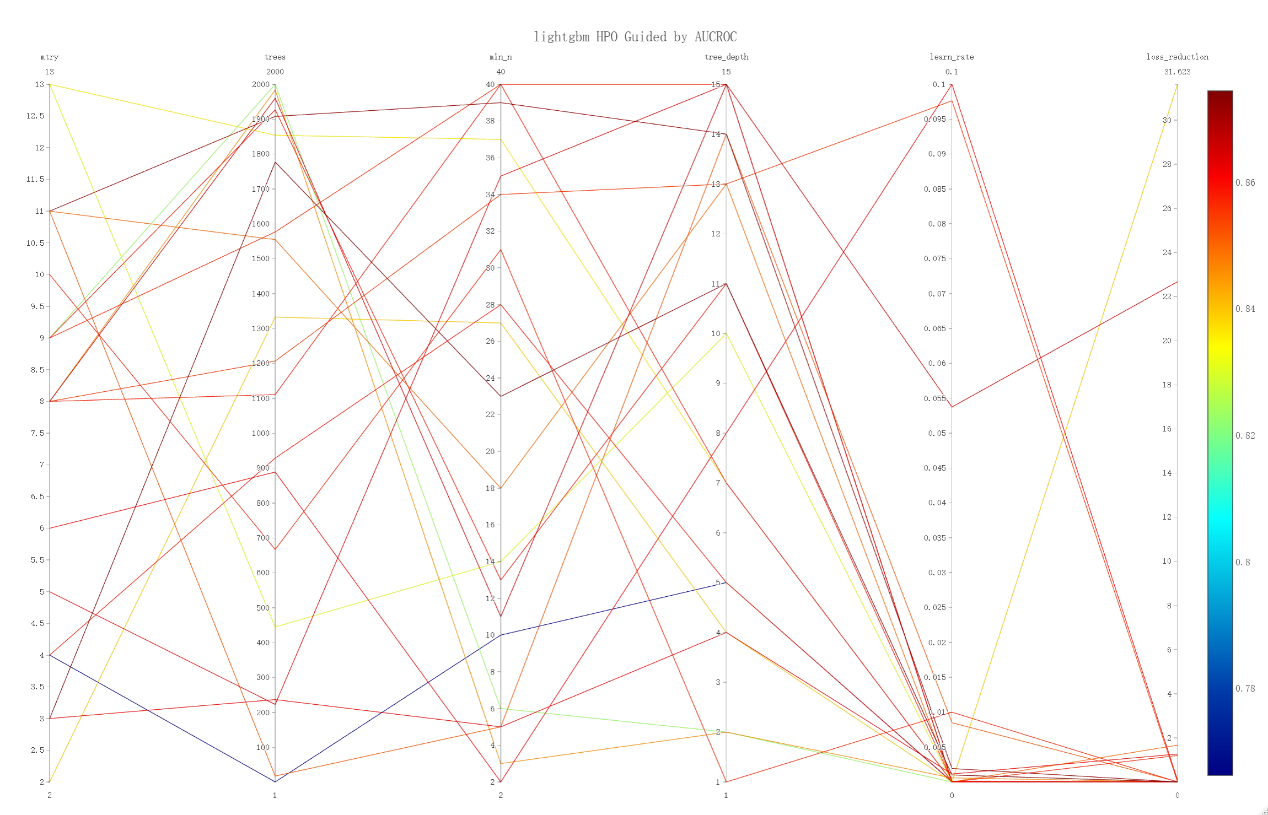
**

**Fig.S5 ROC curve evaluation on the anticoagulant-free set**

**
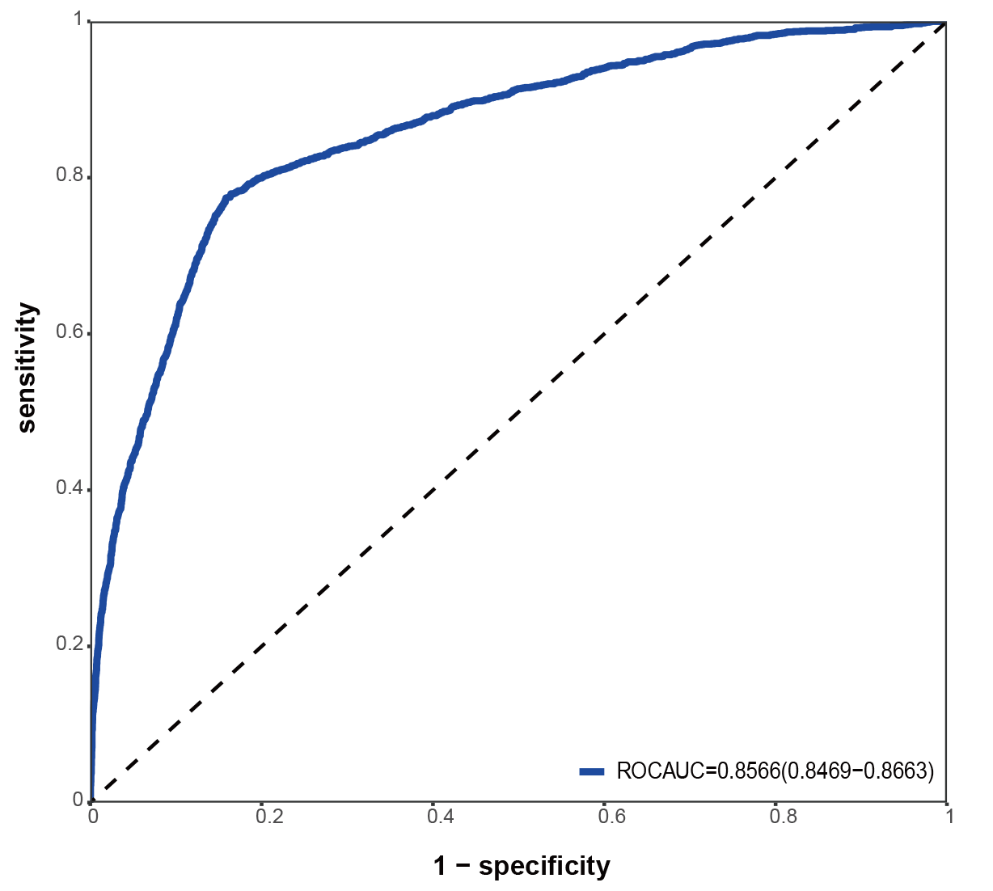
**

**Fig.S6 Confusion matrix on the anticoagulant-free set**

**
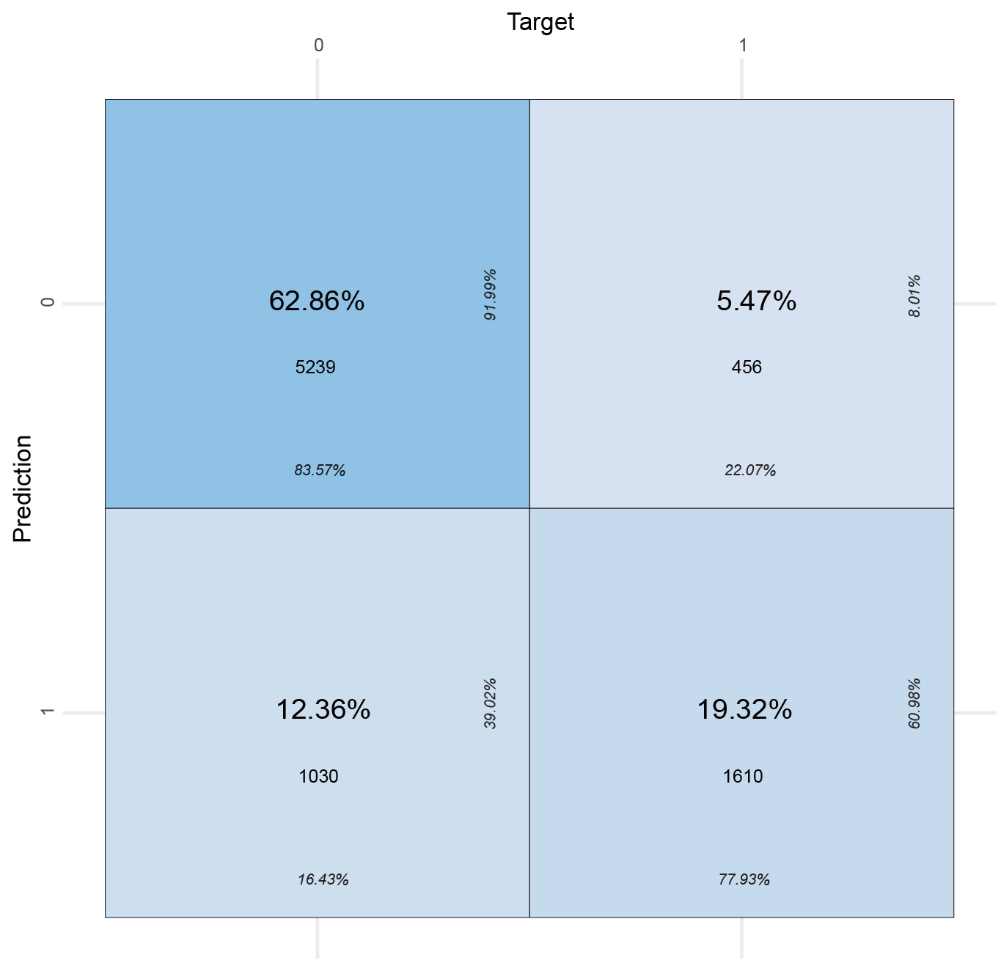
**

**Fig.S7 ROC curve evaluation on the external validation set (eICU-CRD)**

**
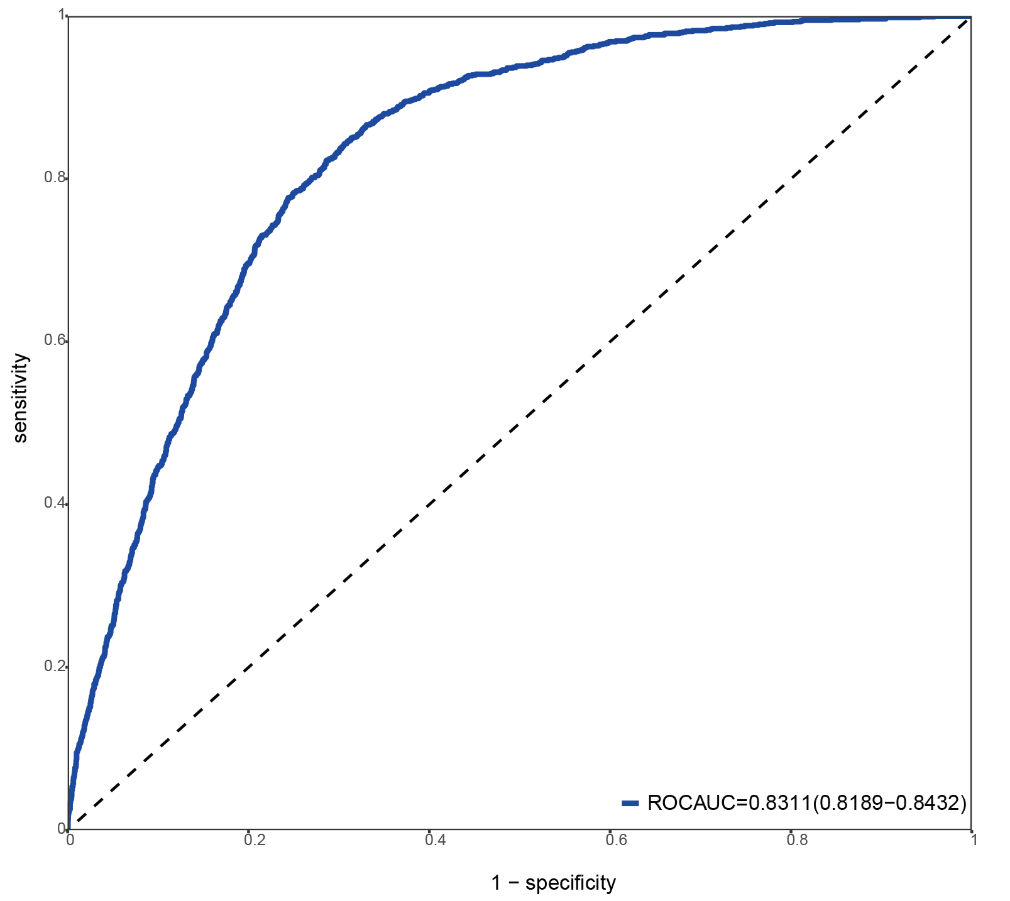
**

**Fig.S8 Confusion matrix on the external validation set (eICU-CRD)**

**
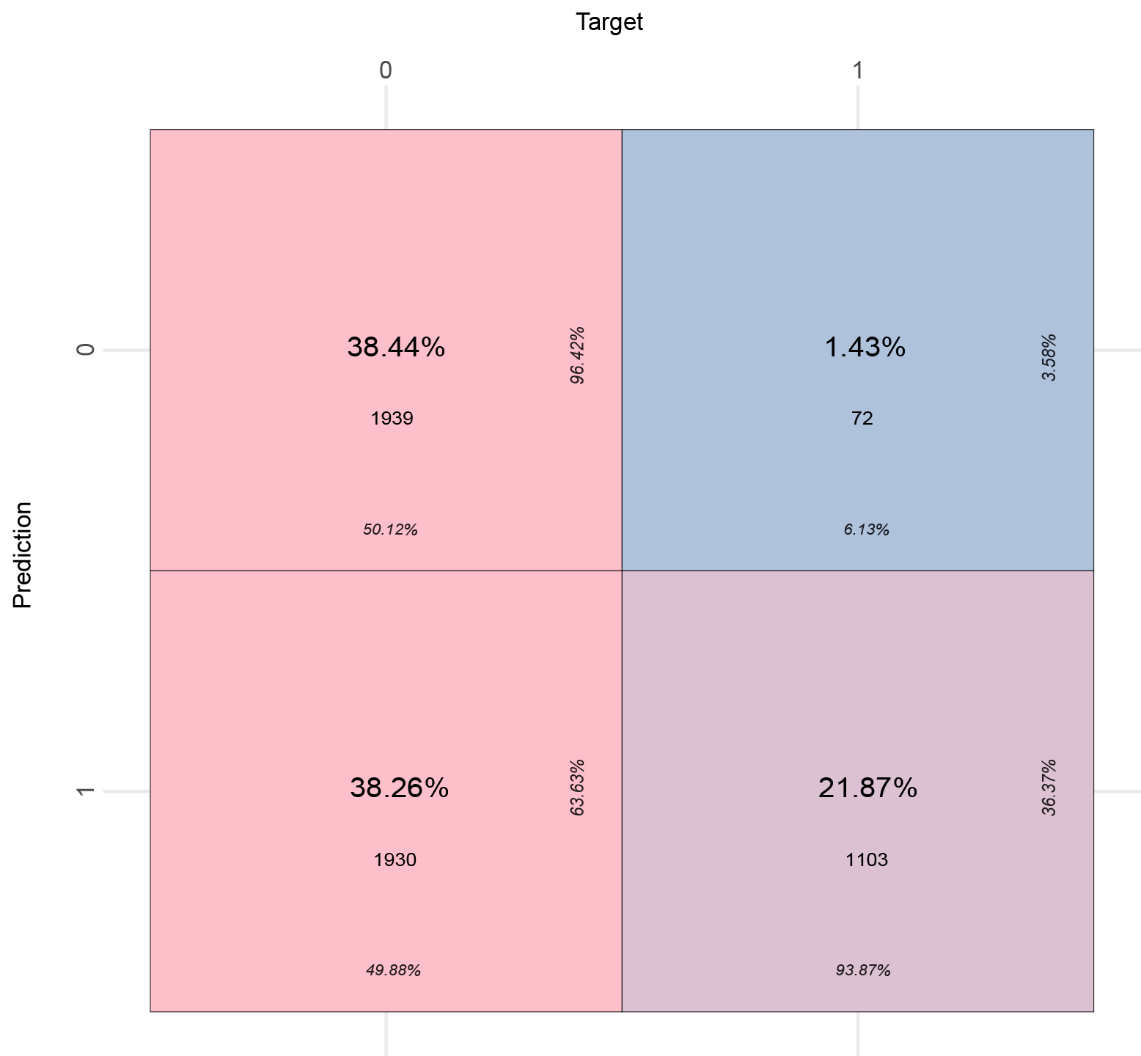
**
